# Identification of genes involved in energy metabolism in preeclampsia and discovery of early biomarkers

**DOI:** 10.3389/fimmu.2025.1496046

**Published:** 2025-02-04

**Authors:** Ruohua Li, Cuixia Zhou, Kejun Ye, Haihui Chen, Mengjia Peng

**Affiliations:** Department of Gynecology and Obstetrics, The Third Affiliated Hospital of Wenzhou Medical University, Rui’an, China

**Keywords:** preeclampsia, energy metabolism, gene expression, biomarkers, immune infiltration

## Abstract

**Background:**

Preeclampsia is a complex pregnancy condition marked by hypertension and organ dysfunction, posing significant risks to maternal and fetal health. This study investigates the role of energy metabolism-associated genes in preeclampsia development and identifies potential early diagnostic biomarkers.

**Methods:**

Preeclampsia datasets from Gene Expression Omnibus were analyzed for batch correction, normalization, and differential expression. Enrichment analyses using gene ontology, Kyoto Encyclopedia of Genes and Genomes, and gene set enrichment were performed. Protein-protein interaction networks were constructed to identify key genes, and regulatory networks involving transcription factors, miRNAs, and RNA-binding proteins were established. Differential expression was validated with receiver operating characteristic curve analyses, and immune infiltration was assessed.

**Results:**

Six energy metabolism-related genes were identified. Enrichment analyses revealed their involvement in glycolysis, gluconeogenesis, lipid transport, bone remodeling, and glucagon secretion. Key differentially expressed genes included *CRH(Corticotropin-Releasing Hormone)*, *LEP(Leptin), PDK4(Pyruvate Dehydrogenase Kinase Isozyme 4)*, *SPP1(Secreted Phosphoprotein 1)*, *and SST(Somatostatin)*. *PDK4* exhibited moderate accuracy in receiver operating characteristic analysis. Immune infiltration analysis indicated significant differences between preeclampsia and control samples. qRT-PCR confirmed *LEP* and *CRH* increased, while *SPP1* expression in preeclampsia samples.

**Conclusion:**

Dysregulated energy metabolism-related genes may contribute to preeclampsia through metabolic and immune changes. Identifying these genes aids in understanding preeclampsia’s molecular basis and early diagnosis. Future studies should validate these markers in larger cohorts and explore targeted treatments.

## 1 Introduction

Preeclampsia (PE) is a complex condition that affects various bodily systems and occurs in 2%–8% of pregnancies worldwide. It continues to be a significant cause of maternal and fetal morbidity and mortality (1). It is believed that PE is responsible for approximately 76,000 maternal and 500,000 fetal fatalities annually (2). It is a pregnancy-related disease that usually occurs after 20 weeks of gestation, characterized by high blood pressure and proteinuria. According to the definition of the World Health Organization, PE refers to a pregnant woman with a blood pressure ≥140/90 mmHg in the later stages of pregnancy and urine containing ≥300 milligrams of protein. It poses significant health hazards, including the potential onset of eclampsia, hemolysis, elevated liver enzymes, and low platelet count (HELLP) syndrome, and enduring cardiovascular issues (3). The pathophysiological mechanism of PE is intricate, encompassing various factors such as placental insufficiency, vascular endothelial dysfunction, and immune dysregulation. Research has demonstrated that in a normal pregnancy, the placenta releases specific signaling molecules to facilitate maternal blood vessel dilation and increased blood flow to accommodate the fetal growth requirements. However, in individuals with PE, there is often inhibition of placental development and function, resulting in damage to endothelial cells and a systemic inflammatory response, ultimately leading to elevated blood pressure and other complications (4). Despite advancements in prenatal care, the primary method for diagnosing PE is to monitor blood pressure and protein levels in the urine. The clinical physician also takes into consideration other potential symptoms, such as cephalalgia, visual impairments, epigastric discomfort, and renal function irregularities. The presence of these symptoms is typically associated with the severity of the condition and holds prognostic significance. Treatment includes using antihypertensive drugs to control the mother’s blood pressure and early low-dose aspirin and calcium supplements to reduce the risk of developing PE. In severe cases, termination of pregnancy is frequently required; however, this generally results in premature birth. Although these interventions are necessary, treatment options for PE are significantly limited, focusing primarily on symptom management rather than addressing the underlying cause of the condition (5). Current treatments have potential limitations, including side effects from antihypertensive drugs and early delivery-associated risks, emphasizing the importance of improving our understanding of PE management strategies.

The disruption of metabolism and metabolites in PE pathogenesis is becoming an essential component of the disease pathophysiology. Studies have reported that carbohydrate and lipid metabolism abnormalities are essential in the etiology and clinical progression of PE (6, 7). Previous studies have indicated a connection between the diverse expression of energy metabolism-related genes (EMRGs) and the emergence of several pregnancy complications, including gestational diabetes mellitus and fetal obesity. This suggests that these conditions can have a common pathophysiological foundation and can be potential targets for treatment (8). Furthermore, differential expression of EMRGs has been associated with altered mitochondrial function and oxidative stress (9), which are characteristic features of the placental pathology in PE (10). Despite the preceding insights, there are significant gaps in understanding the complex energy metabolic pathways and their interactions in PE pathogenesis.

A comprehensive comprehension of the pathogenesis, biomarkers, and associated complications of PE is imperative for enhancing early diagnosis and treatment efficacy. Our study aimed to identify and analyze EMR differentially expressed genes (DEGs) in PE and determine their functional significance. Using bioinformatics methods, including data collection, differential gene expression analysis, functional pathway enrichment, protein-protein interaction (PPI) network creation, regulatory network visualization, and immune infiltration assessment, we provide a new perspective on molecular alterations in PE. This integrated genomic and bioinformatics approach aims to develop novel diagnostic markers and therapeutic targets, increasing our understanding of the molecular foundation underlying PE and aiding in personalized medical strategies to mitigate its impact on mothers and offspring.

## 2 Materials and methods

### 2.1 Data download

The gene expression omnibus (GEO) database (11) (https://www.ncbi.nlm.nih.gov/geo/) provided the PE datasets GSE60438 (12) and GSE75010 (13–18), which were retrieved using the R package “GEOquery”. Dataset GSE60438 was derived from *Homo sapiens*, originating from decidua basalis tissue, with chip platforms GPL10558 and GPL6884. Dataset GSE75010 was derived from *Homo sapiens* placental tissues using the chip platform GPL6244. Detailed information is provided in **Table 1**. Additionally, dataset GSE60438 contained 42 control and 35 PE samples on the GPL10558 platform and 23 control and 25 PE samples on the GPL6884 platform. Dataset GSE75010 comprised 77 control and 80 PE samples. Following batch correction, the combined data from both GPL platforms in dataset GSE60438 were included in the study, whereas dataset GSE75010 was used as a validation set.

**Table 1:** GEO Microarray Chip Information.

	GSE60438	GSE60438	GSE75010
Platform	GPL10558	GPL6884	GPL6244
Species	Homo sapiens	Homo sapiens	Homo sapiens
Tissue	Decidua Basalis	Decidua Basalis	Placenta
Samples in PE group	35	25	80
Samples in Control group	42	23	77
Reference	PMID: 26010865	PMID: 26010865	PMID: 27160201; PMID: 28962696; PMID: 29187609; PMID: 29507646; PMID: 30278173; PMID: 30312585

GEO, Gene Expression Omnibus; PE, Preeclampsia.

We obtained EMRGs from the GeneCards database (19). The GeneCards database provides extensive provides on genes in the human body. After conducting a search using the term “Energy Metabolism” and filtering for “Protein Coding” and “Relevance Score > 2” EMRGs, 571 EMRGs were obtained. Furthermore, using “Energy Metabolism” as the keyword in PubMed, 8 EMRGs were found in the published literature (20). Following the combination and elimination of duplicates, 573 EMRGs were identified, with detailed information presented in **Supplementary Table S1**.

The “sva” (21) package in R was used to correct batch effects in data from two GPL platforms (GPL10558 and GPL6884) to obtain the merged GEO dataset (combined datasets). The combined datasets included 65 control and 60 PE samples. Finally, the annotation and standardization of the merged datasets were performed using the R software package “limma” (22). To determine the impact of the batch effect, we performed a principal component analysis (PCA) (23) on the expression matrix before and following its removal.

### 2.2 Energy metabolism-related differentially expressed genes in PE

The “limma” R package was used to analyze the differences in gene expression between PE and control groups. To identify the DEGs, criteria of |log fold change(logFC)| > 0.5 and a *p* < 0.05 were set. Additionally, genes with a logFC > 0.5 and a *p* < 0.05 were classified as upregulated DEGs. Conversely, genes with a logFC < -0.5 and a *p* < 0.05 were identified as downregulated DEGs. The differential analysis results were depicted using the volcano plot feature provided by the “ggplot2” package in R.

We combined datasets to identify EMRGs that were differentially expressed in association with PE. We determined variance to identify genes exhibiting significant differences (|logFC| > 0.5 and *p* < 0.05). Venn diagrams were used to map the intersection of the DEGs and EMRGs, enabling the identification of EMRDEGs. We generated a heatmap with the R package “pheatmap”. Furthermore, we constructed a chromosome localization map using the R package “RCircos” (24).

### 2.3 Enrichment analysis using gene ontology and the Kyoto encyclopedia of genes and genomes

GO (25) analysis is a widely used methodology for in-depth investigations aimed to improve functionality across multiple dimensions, including biological process (BP), cellular component (CC), and molecular function (MF). The KEGG (26) database is an extensive resource for deciphering the intricate functions and uses of biological systems by connecting genetic information with biochemical pathways and cellular activities. We employed the R package “clusterProfiler” (27) to perform GO and KEGG enrichment analyses on the EMRDEGs. The parameters established for including genes were an adjusted *p*-value (adj. *p*) < 0.05 and a false discovery rate (FDR) < 0.05, both of which were considered statistically significant. The Benjamini–Hochberg (BH) procedure was used as a *p*-value adjustment method.

### 2.4 Gene set enrichment analysis

GSEA (28) is a statistical technique to determine if predefined gene groups exhibit significant enrichment across various biological conditions. In this study, the genes of combined datasets were first sorted according to logFC values. Then, GSEA was performed on the entire set of genes from the merged datasets, using the “clusterProfiler” package in R. GSEA settings were accessing the “c2.cp.all.v2022.1.Hs.symbols.gmt [All Canonical Pathways] (3050)” gene set from the Molecular Signatures database (29), using 2022 seeds, performing 1000 calculations, with each gene set containing between 10 and 500 genes. The evaluation standards were established as adj. *p* < 0.05 and FDR (q-value) < 0.05 via the BH method for *p*-value adjustment.

### 2.5 Analysis of PPI and identification of key genes

The PPI network includes essential proteins involved in numerous biological functions, including signaling pathways, gene expression regulation, metabolism of energy and substances, and cell cycle management. This network is crucial for comprehending protein functionalities, signaling mechanisms, and physiological and pathological functional associations. The search tool for the retrieval of interacting genes/proteins (STRING) database (30) (https://cn.string-db.org/) investigates the connections among identified and anticipated proteins. This study used the STRING database to build a PPI network associated with EMRDEGs, adhering to the criteria of a minimum interaction coefficient exceeding 0.400, which corresponded to a medium confidence level. The associated regions within the PPI network could indicate molecular assemblies with distinct biological roles. Certain genes were identified as key genes within the PPI network as a result of their interactions with other genes.

The GeneMANIA database (31) (https://genemania.org/) was used to predict potential gene functions, evaluate gene lists, and pinpoint genes for further functional analyses. When provided with a list of query genes, GeneMANIA identifies functionally similar genes by analyzing a comprehensive genomics and proteomics dataset. It assigns weights to each functional genomic dataset based on the anticipated value of the query in this process. Besides, GeneMANIA can predict gene functions by identifying genes likely to share tasks with a given query gene based on their interactions. Using the GeneMANIA online website, the PPI network was created to predict genes with functions similar to those of key genes.

### 2.6 Construction of regulatory network

Gene expression is regulated by transcription factors (TFs) through their interaction with crucial genes during the post-transcriptional phase. We used the ChIPBase database (http://rna.sysu.edu.cn/chipbase/) (32) to obtain data on TFs and determine their control over essential genes. The screening criterion for mRNA-TF interaction pairs was based on the total number of upstream and downstream samples, which was required to be > 5. Finally, the mRNA-TF regulatory network was developed using Cytoscape software.

The role of miRNA in regulation is vital for developmental and evolutionary mechanisms in organisms. Different target genes could be regulated, and several miRNAs could influence a single target gene. To investigate the association between pivotal genes and miRNA, we retrieved the miRNA that interacted with key genes from the encyclopedia of RNA interactomes (ENCORI) database (https://rnasysu.com/encori/) (33). We used a screening threshold of pancancerNum > 5 to select mRNA-miRNA interaction pairs. The interaction network between mRNA and miRNA was illustrated using Cytoscape software.

Furthermore, RNA-binding proteins (RBPs) control gene expression by engaging with crucial mRNAs after transcription. We used the ENCORI database to extract RBP information and analyze their regulation of key mRNAs.The criterion for screening mRNA-RBP interaction pairs was clusterNum >1. Ultimately, Cytoscape software was used to visualize the constructed mRNA-RBP regulatory network.

### 2.7 Validation of differential gene expression and analysis of key genes using receiver operating characteristic curves

To analyze the variation in key gene expression between the PE and control groups within the combined datasets, we used the Mann–Whitney U test. Comparative maps were constructed based on the expression levels of these essential genes. Subsequently, the R package “pROC” (34) was used to generate the ROC curve for the significant genes. The area under the curve (AUC) evaluated the effectiveness of gene expression in diagnosing PE. The validation process was performed using the GSE75010 dataset.

### 2.8 Immune infiltration analysis

We measured the proportion of immune cell infiltration using single-sample (ss) GSEA (35). The recognized categories of immune cells comprised activated CD8 + T cells, activated dendritic cells, gamma-delta T cells, natural killer (NK) cells, regulatory T cells (Tregs), and several other human immune cell subtypes. The proportion calculated through ssGSEA was used to illustrate the relative levels of immune cell infiltration in each sample, creating an immune cell infiltration matrix. Then, immune cells indicating significant variations between the two groups were selected for additional analysis, and their relationships were evaluated using the Spearman method. Correlation heatmaps were created with the R package “pheatmap” to demonstrate the correlation between immune cells. The Spearman method determined the relationship between crucial genes and immune cells, with a significance threshold set at *p* < 0.05. Using the R package “ggplot2”, a bubble map was drawn to illustrate the connection between essential genes and immune cells. We selected immune cells with top1 positive and top1 negative correlation with key genes and plotted correlation scatter plots using ggplot2.

### 2.9 Patient and tissue samples

Placenta samples were obtained from 52 pregnant women who underwent cesarean sections at the Third Affiliated Hospital of Wenzhou Medical University. 26 had PE, and 26 were healthy controls matched for gestational age. Each group included 14 term pregnancy and 12 preterm pregnancies. Ethical approval was obtained from the Research Ethics Committee at the Ruian People’s Hospital, under approval number YJ2024130. All participants provided written consent. The inclusion criteria for the PE group included blood pressure ≥ 140/90 mmHg and 24-h urinary protein ≥ 0.3 g/24 h after 20 weeks of gestation, age between 20 and 40 years old, and no significant abnormalities during pregnancy. Exclusion criteria included other pregnancy complications such as gestational diabetes mellitus; Prepregnancy comorbidities such as prepregnancy hypertension, prepregnancy diabetes, serious medical and surgical diseases, infectious diseases such as COVID-19, obstetric complications, congenital diseases of the fetus, or the use of drugs that may affect the results of the experiment. After delivery, a tissue sample was extracted from the central region of the placenta and preserved at –80°C for long-term storage.

### 2.10 Isolation of RNA and analysis using quantitative real-time-polymerase chain reaction

Total RNA was extracted from placental tissue samples using the tissue total RNA isolation kit V2 (Vazyme) according to the manufacturer’s instructions. The concentration and purity of the extracted RNA were assessed using a NanoDrop spectrophotometer (Thermo Fisher Scientific). RNA samples with an A260/A280 ratio between 1.8 and 2.0 were considered suitable for further analysis. Subsequently, 1 µg of total RNA was reverse transcribed into complementary DNA (cDNA) using the HiScript III All-in-one RT SuperMix (Vazyme) in a 20 µL reaction volume. The reverse transcription reaction was performed at 25°C for 5 minutes, followed by 50°C for 15 minutes, and terminated by heating at 85°C for 5 minutes. Quantitative real-time PCR (qRT-PCR) was conducted using the CFX Connect real-time PCR system (BioRad, Hercules, CA, USA) with Taq Pro Universal SYBR qPCR Master Mix (Vazyme). Each qRT-PCR reaction was carried out in a 10 µL volume containing 5 µL of SYBR Green Master Mix, 0.5 µL of each forward and reverse primer (10 µM), 1 µL of cDNA template, and 3 µL of nuclease-free water. The thermal cycling conditions were as follows: initial denaturation at 95°C for 30 seconds, followed by 40 cycles of denaturation at 95°C for 10 seconds, annealing at 60°C for 30 seconds, and extension at 72°C for 30 seconds. A melt curve analysis was performed to verify the specificity of the amplification products. The relative expression levels of the key genes were normalized to the expression of the housekeeping gene glyceraldehyde-3-phosphate dehydrogenase (GAPDH) using the 2−^ΔΔCt^ method. All reactions were performed in triplicate, and the average Ct values were used for analysis. The results were expressed as fold changes in gene expression relative to the control group.

### 2.11 Isolation of protein and analysis using Western Blotting

Tissues were minced and homogenized in RIPA lysis buffer (P0013B, Beyotime) with PMSF (100 mM, ST506, Beyotime), using 150-250 µL of lysis buffer per 20 mg of tissue. The homogenates were centrifuged similarly to obtain the supernatant. Protein concentration was determined using the BCA protein assay kit. Samples were diluted to equal concentrations with RIPA lysis buffer containing PMSF and mixed with 5× protein loading buffer. The samples were denatured by heating at 100°C for 5-10 minutes and then cooled on ice. SDS-PAGE was performed using self-prepared gels by first casting the separating and stacking gels between clean glass plates. The samples were then loaded into the wells formed by the comb in the stacking gel, and electrophoresis was conducted to separate proteins based on their molecular weight. Proteins were transferred to PVDF membranes (ISEQ00010, Millipore) using a wet transfer system (Mini Trans-Blot, BIO-RAD). The membranes were blocked with non-fat milk blocking solution for 1-2 hours at room temperature and incubated overnight at 4°C with primary antibodies diluted in antibody dilution buffer (P0256-500ml, Beyotime). After washing, the membranes were incubated with HRP-conjugated secondary antibodies for 1 hour at room temperature. The protein bands were visualized by using BeyoECL Plus working solution (P0018S, Beyotime) and detected with a chemiluminescence imaging system. The relative expression levels of proteins were analyzed by Image J.

### 2.12 Statistical analysis

The study used R software (version 4.3.1) for statistical analysis and data handling. The independent student t-test was implemented to assess the statistical significance of normally distributed data and compare continuous variables between two groups unless otherwise specified. For non-normally distributed variables, the Mann–Whitney U or Wilcoxon rank sum test was used to determine differences. Furthermore, the Kruskal–Wallis test was applied to compare outcomes among three or more groups. Spearman’s rank correlation was used to determine the correlation coefficients for several molecules without particular specifications. All statistical analyses used two-tailed *p*-values, with a significance level set at *p* < 0.05.

## 3 Results

### 3.1 Analytical flow diagram

**Figure 1:**
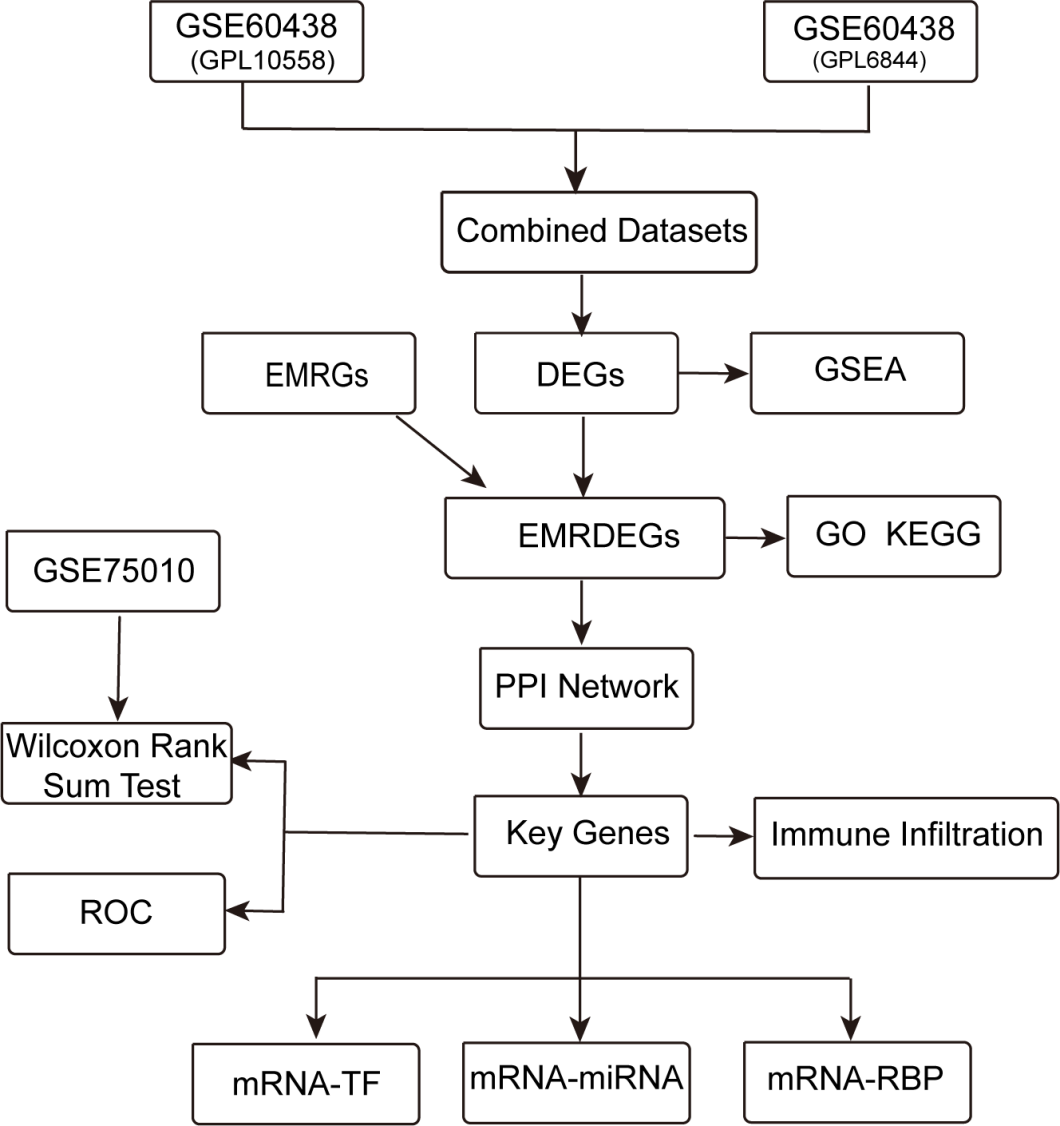
Technology roadmap. DEGs, Differentially Expressed Genes; EMRGs, Energy Metabolism-Related Genes. EMRDEGs, Energy Metabolism-Related Differentially Expressed Genes; GO, Gene Ontology; KEGG, Kyoto Encyclopedia of Genes and Genomes; GSEA, Gene Set Enrichment Analysis; ROC, Receiver Operating Characteristic Curve; PPI Network, Protein-protein Interaction Network; TF, Transcription Factor; RBP, RNA-Binding Protein.

**Figure 1** displays the technical approach of the study, providing a concise overview of the analytical processes used in this study.It begins with the combination of datasets GSE60438 (GPL10558 and GPL6884) and proceeds with the identification of differentially expressed genes (DEGs). Energy metabolism-related genes (EMRGs) are intersected with DEGs to identify energy metabolism-related differentially expressed genes (EMRDEGs). The subsequent enrichment analyses include Gene Set Enrichment Analysis (GSEA), Gene Ontology (GO), and Kyoto Encyclopedia of Genes and Genomes (KEGG) enrichment. A protein-protein interaction (PPI) network is constructed to identify key genes, which are further analyzed for immune infiltration. Key genes are validated using the GSE75010 dataset through the Wilcoxon Rank Sum Test and Receiver Operating Characteristic (ROC) analysis. Finally, regulatory networks involving mRNA-TF, mRNA-miRNA, and mRNA-RBP interactions are constructed to understand the regulatory mechanisms.

### 3.2 Merging of PE datasets

**Figure 2:**
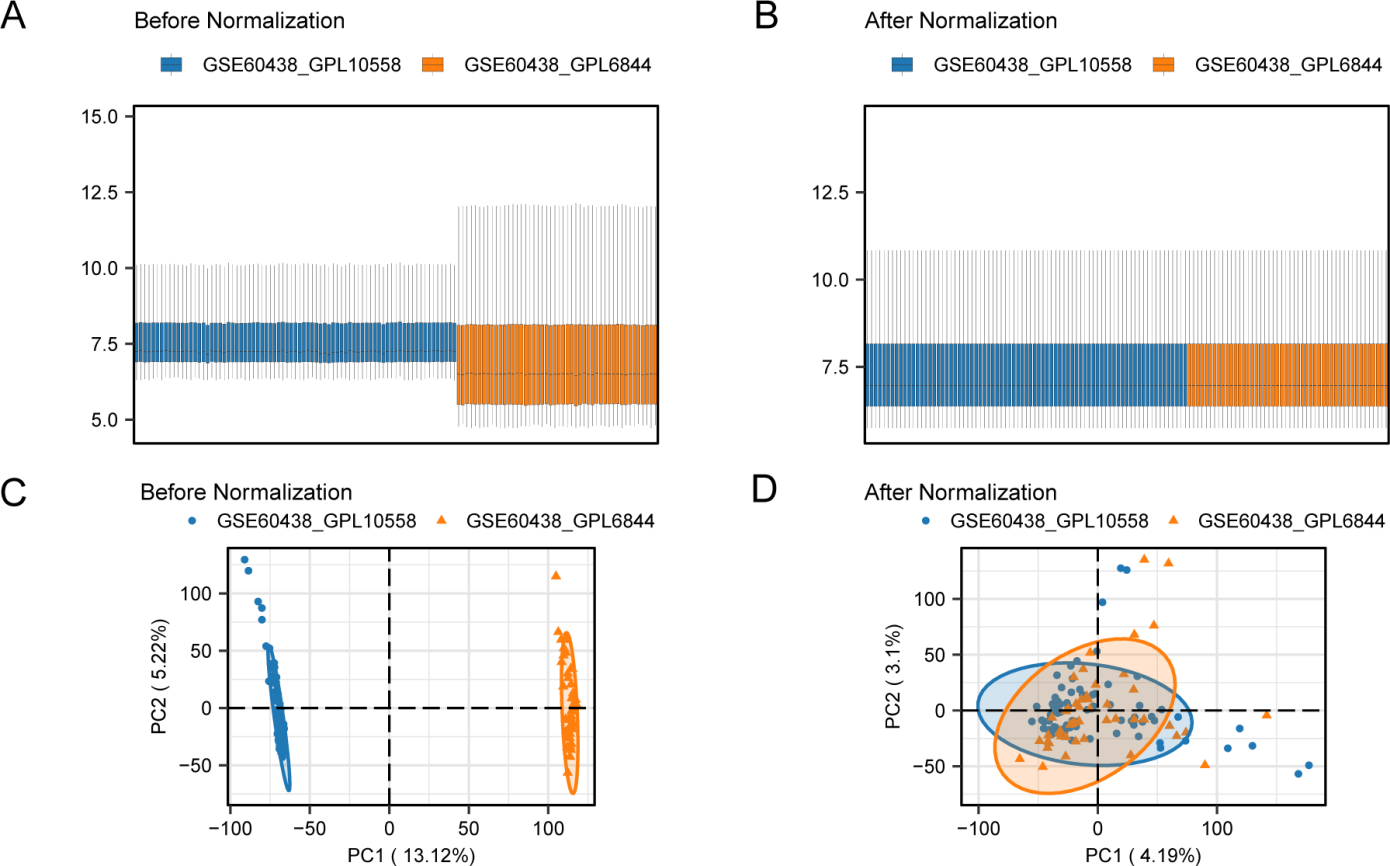
Batch effects removal of GSE60438 (GPL10558, GPL6884). **(A)** Boxplots of combined datasets distribution before batch removal. **(B)** Post-batch integrated combined datasets distribution boxplots. **(C)** PCA plot of the datasets before debatching. **(D)** Go to the PCA map of the combined datasets after batch processing. PCA, Principal Component Analysis; PE, Preeclampsia. The PE dataset GSE60438 (GPL10558 platform) is blue, and the PE dataset GSE60438 (GPL6884 platform) is orange.

To eliminate batch effects from the PE datasets GSE60438 (using GPL10558 and GPL6884 platforms), the R package “sva” was used, resulting in combined datasets. Boxplots (**Figures 2A, B**) were used to compare the expression values of the datasets pre- and post-batch effect removal. Furthermore, a PCA plot (**Figures 2C, D**) compared the distribution of low-dimensional features in the dataset before and after addressing batch effects. The outcomes from the distribution box plot and PCA plot indicated that the batch effect in the PE dataset samples was significantly reduced after batch correction.

### 3.3 Genes with altered expression associated with energy metabolism in PE

**Figure 3:**
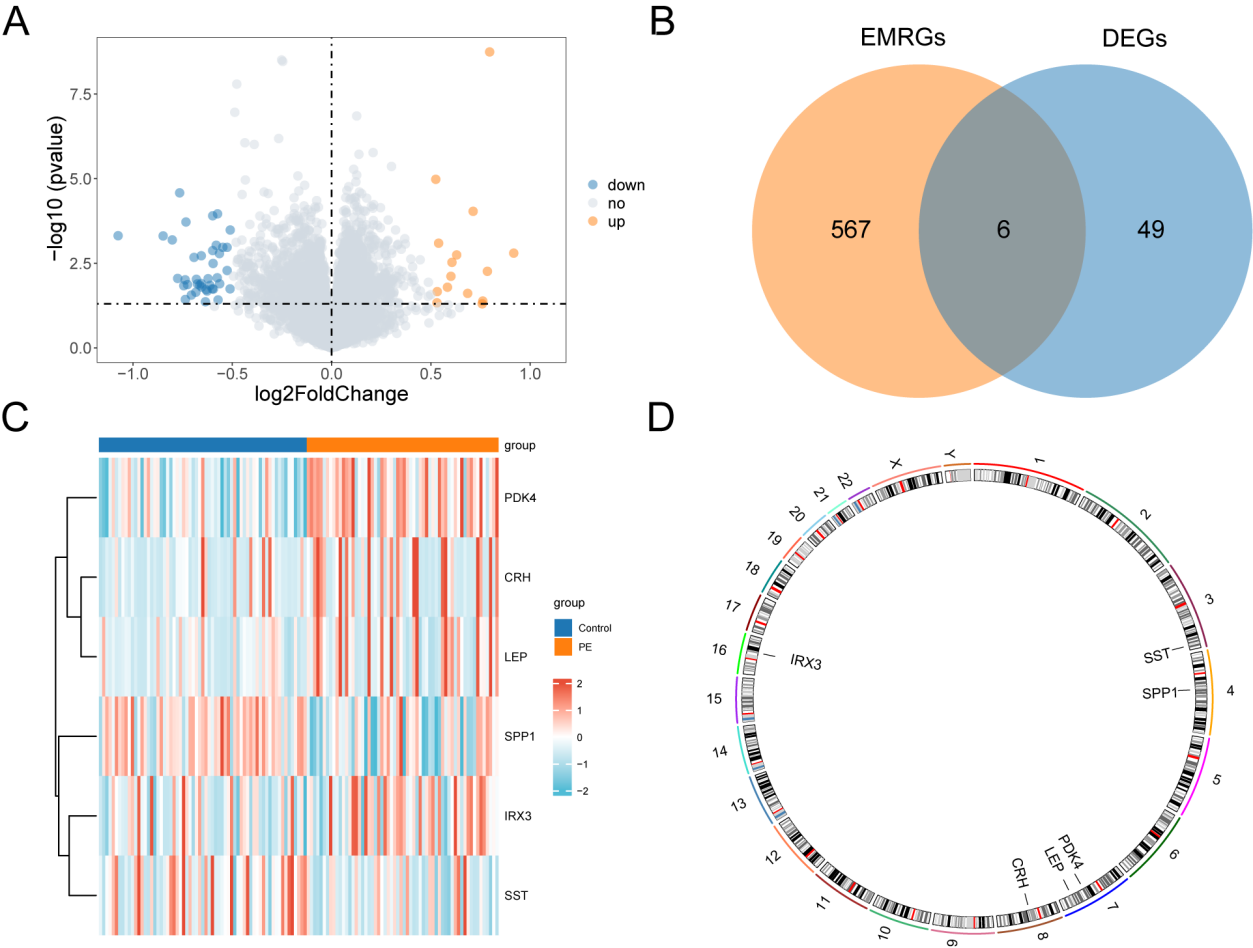
Differential gene expression analysis. **(A)** Volcano plot of differentially expressed genes analysis between PE and Control groups in combined datasets. **(B)** DEGs and EMRGs Venn diagram in the combined datasets. **(C)** Heat map of EMRDEGs in the combined datasets. **(D)** Chromosomal mapping of EMRDEGs; DEGs, Differentially Expressed Genes; EMRGs, Energy Metabolism Related Genes; EMRDEGs, Energy Metabolism Related Differentially Expressed Genes. The orange is the PE group, and the blue is the Control group. The red in the heat map represents high expression, and the blue represents low expression.

The data from the combined datasets were separated into PE and control groups. We performed a comparative analysis of gene expression levels between PE and control groups across the combined datasets using the R package “limma”. The findings identified 55 genes with differential expression, satisfying the criteria of |logFC| > 0.5 and a *p* < 0.05 in the combined datasets. Out of these DEGs, 15 indicated increased expression (logFC > 0.5, *p* < 0.05), whereas 40 exhibited decreased expression (logFC < –0.5, *p* < 0.05), which was illustrated in the volcano plot analysis of the dataset (**Figure 3A**).

To identify genes that exhibited differential expression and were associated with energy metabolism, we selected genes with |logFC| > 0.5 and a *p* < 0.05 from the overlap of DEGs and EMRGs (**Figure 3B**). Six EMRDEGs, including *CRH*, *IRX3(Iroquois Homeobox 3)*, *LEP*, *PDK4*, *SPP1*, and *SST*, were identified (**Table 2**). The variations in the expression of identified EMRDEGs among different sample groups in the combined datasets were investigated through the intersection results. The analysis results were visualized in a heatmap created with the “pheatmap” package in R (**Figure 3C**). Besides, the R package “RCircos” was used to plot the positions of these six EMRDEGs on human chromosomes and constructed a chromosome localization map (**Figure 3D**). The mapping revealed that most of these EMRDEGs were located on chromosome 7, particularly *LEP* and *PDK4*.

**Table 2:** Description of EMRDEGs.

ID	Description	logFC	AveExpr	t	*p*-value	B
CRH	Corticotropin Releasing Hormone	0.917196	7.729465	3.229391	0.001586	1.28882
PDK4	Pyruvate Dehydrogenase Kinase 4	0.795918	9.086324	6.492432	1.80 e-09	11.111
SPP1	Secreted Phosphoprotein 1	0.76471	12.14558	4.36649	2.62 e-05	2.371449
SST	Somatostatin	0.56438	7.620709	3.22046	0.001632	1.31405
LEP	Leptin	0.539052	7.895897	3.434848	0.000805	0.69233

EMRDEGs, Energy Metabolism-Related Differentially Expressed Genes.

### 3.4 Enrichment analysis using GO and KEGG

**Figure 4:**
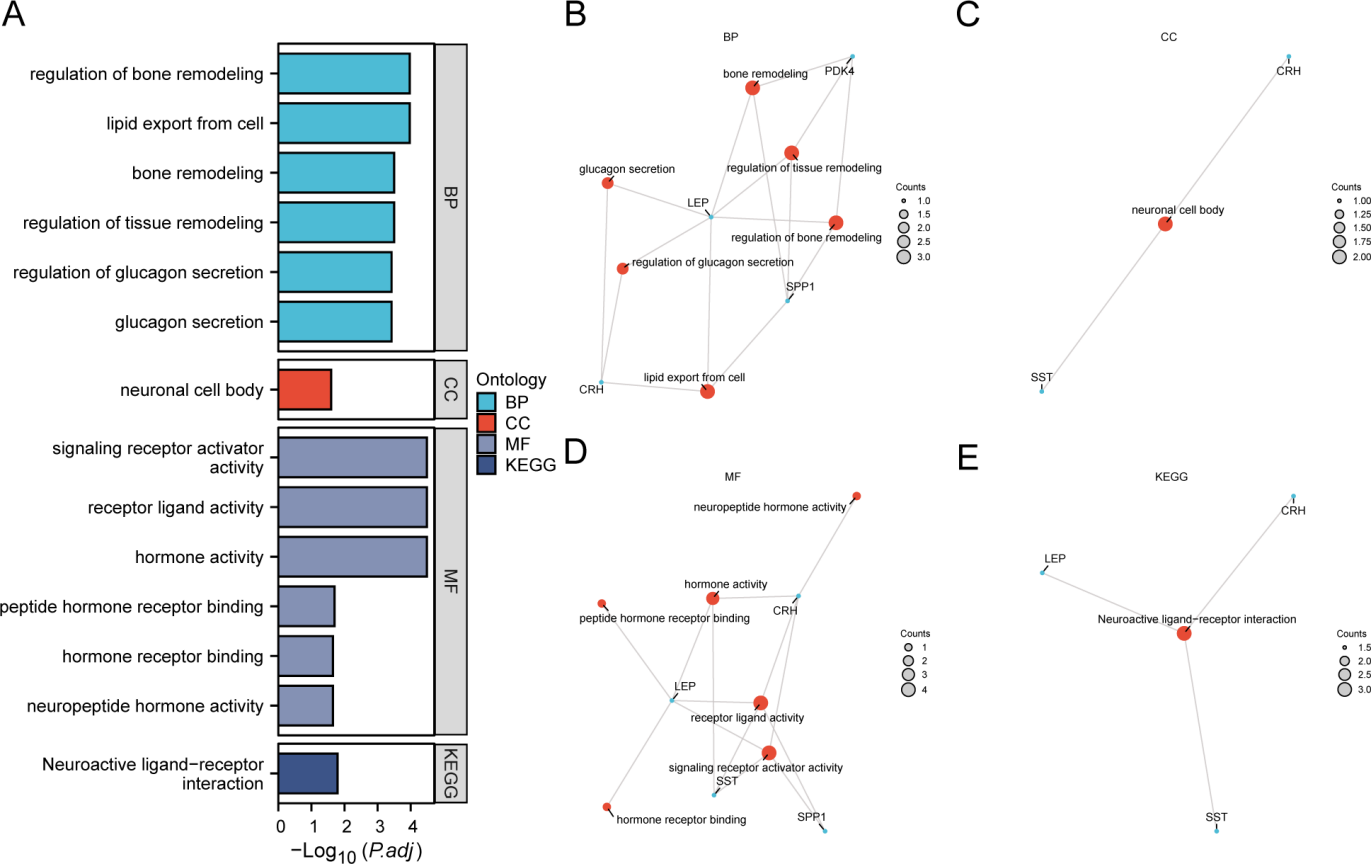
GO and KEGG enrichment analysis for EMRDEGs. **(A)** Bar graph of GO and KEGG enrichment analysis results of EMRDEGs: BP, CC, MF, and KEGG. GO terms and KEGG terms are indicated on the ordinate. B-E. GO and KEGG enrichment analysis results of EMRDEGs network diagram exhibiting BP **(B)**, CC **(C)**, MF **(D)**, and KEGG **(E)**. The orange nodes represent items, the blue nodes represent molecules, and the lines represent the relationship between items and molecules. EMRDEGs, Energy Metabolism-Related Differentially Expressed Genes; GO, Gene Ontology; KEGG, Kyoto Encyclopedia of Genes and Genomes; BP, Biological Process; CC, Cellular Component; MF, Molecular Function. The screening criteria for GO and KEGG enrichment analysis were adj. *p* < 0.05, and FDR value (q-value) < 0.05, and the p-value correction method was Benjamini-Hochberg (BH).

We employed GO and KEGG enrichment analyses to investigate the association between BP, CC, MF, and biological pathways (KEGG) of six EMRDEGs and PE. The six EMRDEGs underwent GO and KEGG enrichment analyses (**Table 3**). The results indicated that the six EMRDEGs were primarily involved in several BP, including cell lipid export, regulation of bone remodeling, tissue and bone restructuring, and glucagon release. Furthermore, they were associated with CC, neuronal cell bodies, and MFs related to hormone activity, receptor signaling activation, peptide hormone receptor binding, neuropeptide hormone activity, and other hormone receptor interactions. Additionally, the biological pathway associated with neuroactive ligand-receptor interaction (KEGG) exhibited an increase. The findings from GO and KEGG enrichment analyses were presented using bar graphs (**Figure 4A**).

**Table 3:** Results of GO and KEGG enrichment analysis for EMRDEGs.

ONTOLOGY	ID	Description	GeneRatio	BgRatio	*p*-value	p.adjust	q-value
BP	GO: 0031667	response to nutrient levels	4/6	446/18800	4.5143 e-06	0.00037793	0.0001242
BP	GO: 0009991	response to extracellular stimulus	4/6	479/18800	5.9945 e-06	0.00038785	0.00012746
BP	GO: 0140353	lipid export from cell	3/6	43/18800	2.2184 e-07	0.00010706	3.5186 e-05
BP	GO: 0046850	regulation of bone remodeling	3/6	49/18800	3.3095 e-07	0.00010706	3.5186 e-05
BP	GO: 0034103	regulation of tissue remodeling	3/6	86/18800	1.8302 e-06	0.00031735	0.0001043
BP	GO: 0046849	bone remodeling	3/6	88/18800	1.962 e-06	0.00031735	0.0001043
BP	GO: 0032368	regulation of lipid transport	3/6	118/18800	4.7551 e-06	0.00037793	0.0001242
BP	GO: 0046887	positive regulation of hormone secretion	3/6	122/18800	5.2572 e-06	0.00037793	0.0001242
BP	GO: 0007584	response to nutrient	3/6	150/18800	9.7837 e-06	0.00057546	0.00018912
BP	GO: 1905952	regulation of lipid localization	3/6	155/18800	1.0796 e-05	0.00058206	0.00019129
BP	GO: 0048771	tissue remodeling	3/6	174/18800	1.527 e-05	0.00075997	0.00024976
BP	GO: 0007565	female pregnancy	3/6	185/18800	1.8347 e-05	0.00084791	0.00027866
BP	GO: 0070091	glucagon secretion	2/6	10/18800	3.8155 e-06	0.00037793	0.0001242
BP	GO: 0070092	regulation of glucagon secretion	2/6	10/18800	3.8155 e-06	0.00037793	0.0001242
CC	GO: 0043025	neuronal cell body	2/6	482/19594	0.00848281	0.02544843	0.01785855
MF	GO: 0048018	receptor ligand activity	4/6	489/18410	7.0699 e-06	3.2414 e-05	1.3123 e-05
MF	GO: 0030546	signaling receptor activator activity	4/6	496/18410	7.4802 e-06	3.2414 e-05	1.3123 e-05
MF	GO: 0005179	hormone activity	3/6	122/18410	5.5967 e-06	3.2414 e-05	1.3123 e-05
MF	GO: 0051428	peptide hormone receptor binding	1/6	19/18410	0.00617717	0.0200758	0.00812785
MF	GO: 0005184	neuropeptide hormone activity	1/6	30/18410	0.00973887	0.02250149	0.00910992
MF	GO: 0051427	hormone receptor binding	1/6	32/18410	0.0103853	0.02250149	0.00910992
MF	GO: 0050840	extracellular matrix binding	1/6	55/18410	0.01779409	0.03304617	0.01337902
KEGG	hsa04080	Neuroactive ligand-receptor interaction	3/5	362/8164	0.00080884	0.01617674	0.0119197

GO, Gene Ontology; BP, Biological Process; CC, Cellular Component; MF, Molecular Function; KEGG, Kyoto Encyclopedia of Genes and Genomes; EMRDEGs, Energy Metabolism-Related Differentially Expressed Genes.

Following GO and KEGG enrichment analyses, BP, CC, MF, and biological pathways (KEGG) were schematically presented (**Figures 4B–E**). The connections display the molecules corresponding to the entries, with annotations for each. The magnitude of the nodes indicates the quantity of molecules present in each record.

### 3.5 GSEA

**Figure 5:**
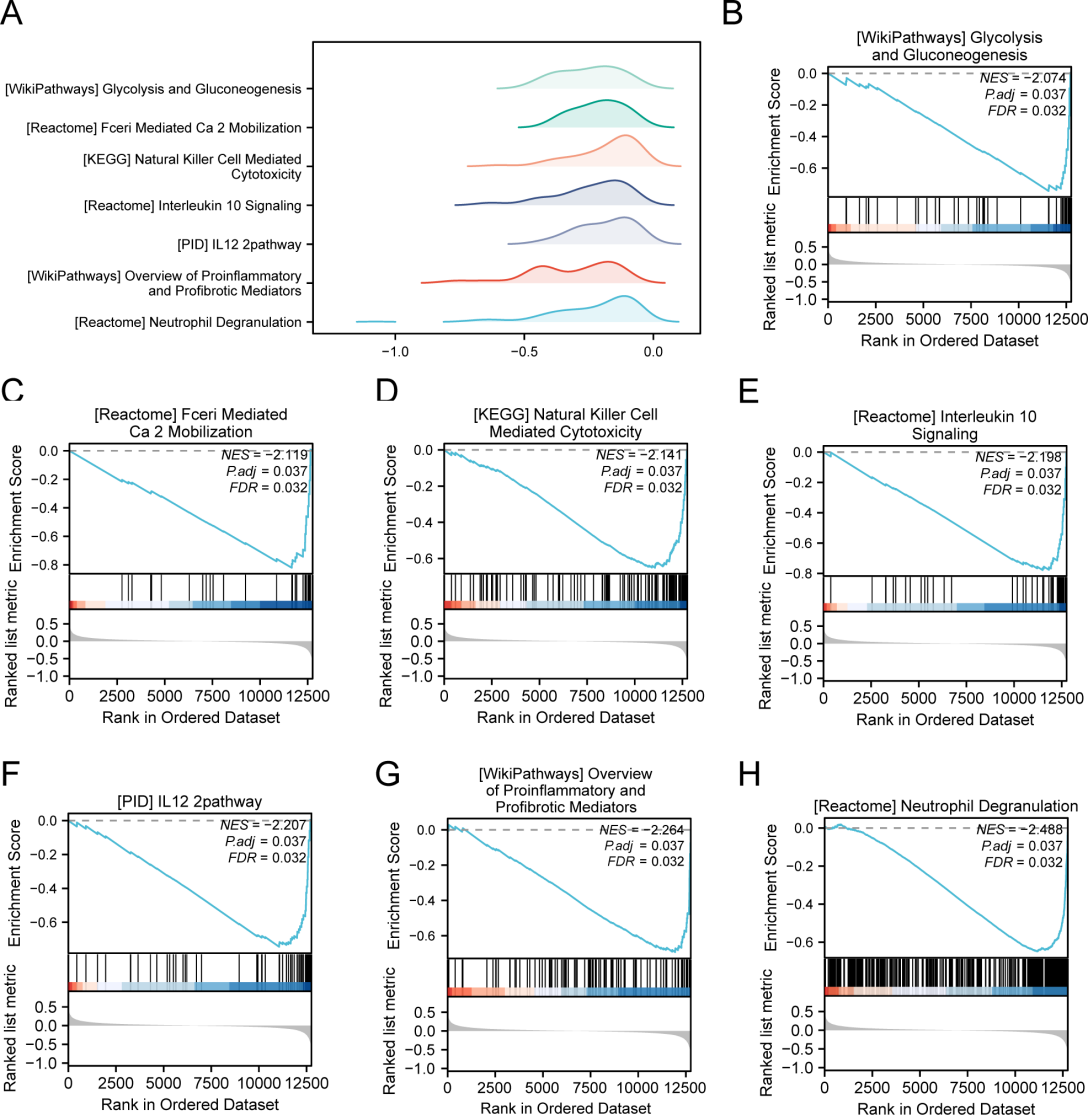
GSEA for combined datasets. **(A)** GSEA mountain map presentation of 7 biological functions of the combined datasets. B-h. GSEA revealed that EMRDEGs were significantly enriched in WP_GLYCOLYSIS_AND_GLUCONEOGENESIS **(B)**, REACTOME_FCERI_MEDIATED_CA_2_MOBILIZATION **(C)**, KEGG_NATURAL_KILLER_CELL_MEDIATED_CYTOTOXICITY **(D)**, Reactome_interleukin_10 signaling **(E)**, PID_IL12_2PATHWAY **(F)**, WP_OVERVIEW_OF_PROINFLAMMATORY_AND_PROFIBROTIC_MEDIATORS **(G)**, REACTOME_NEUTROPHIL_DEGRANULATION **(H)**. GSEA, Gene Set Enrichment Analysis; EMRDEGs, Energy Metabolism-Related Differentially Expressed Genes; The screening criteria of GSEA were adj. *p* < 0.05 and FDR value (q-value) < 0.05, and the p-value correction method was Benjamini-Hochberg (BH).

We conducted GSEA to determine how gene expression levels across the combined datasets influenced PE and to identify the associated BPs. The relationship between affected CCs and performed MFs is depicted in **Figure 5A**, with specific results provided in **Table 4**. The findings indicated that all genes in the combined datasets were significantly enriched in glycolysis and gluconeogenesis (**Figure 5B**), faerie-mediated Ca^2+^ mobilization (**Figure 5C**), NK cell-mediated cytotoxicity (**Figure 5D**), interleukin (IL) 10 signaling (**Figure 5E**), IL12 pathway (**Figure 5F**), an overview of proinflammatory and profibrotic mediators (**Figure 5G**), neutrophil degranulation (**Figure 5H**), and other biologically related functions and signaling pathways.

**Table 4:** Results of GSEA for combined datasets.

ID	Set Size	Enrichment Score	NES	*p*-value	p adjust	q-value
REACTOME_NEUTROPHIL_DEGRANULATION	382	0.64867	2.48836	0.001495	0.036503	0.031576
WP_OVERVIEW_OF_PROINFLAMMATORY_AND_PROFIBROTIC_MEDIATORS	101	0.69267	2.26388	0.001709	0.036503	0.031576
PID_IL12_2PATHWAY	54	0.74499	2.20711	0.001862	0.036503	0.031576
REACTOME_INTERLEUKIN_10_SIGNALING	43	0.77819	2.19766	0.001873	0.036503	0.031576
KEGG_NATURAL_KILLER_CELL_MEDIATED_CYTOTOXICITY	103	0.6511	2.14121	0.001692	0.036503	0.031576
REACTOME_FCERI_MEDIATED_CA_2_MOBILIZATION	29	0.82047	2.11928	0.001946	0.036503	0.031576
WP_GLYCOLYSIS_AND_GLUCONEOGENESIS	40	0.74474	2.07394	0.001905	0.036503	0.031576
PID_IL12_STAT4_PATHWAY	31	0.77487	2.06097	0.001901	0.036503	0.031576
REACTOME_COSTIMULATION_BY_THE_CD28_FAMILY	58	0.65126	1.9598	0.001818	0.036503	0.031576
BIOCARTA_IL17_PATHWAY	13	0.8883	1.95493	0.002066	0.036503	0.031576
PID_IL8_CXCR2_PATHWAY	29	0.75551	1.95148	0.001946	0.036503	0.031576
PID_IL23_PATHWAY	35	0.70314	1.92153	0.001869	0.036503	0.031576
BIOCARTA_NO2IL12_PATHWAY	14	0.85133	1.91891	0.002024	0.036503	0.031576
REACTOME_SIGNALING_BY_INTERLEUKINS	394	0.49928	1.91331	0.001522	0.036503	0.031576
WP_IL1_AND_MEGAKARYOCYTES_IN_OBESITY	22	0.75449	1.86687	0.001931	0.036503	0.031576
REACTOME_MET_PROMOTES_CELL_MOTILITY	40	0.640364	1.8563	0.002096	0.036503	0.031576
REACTOME_GLYCOLYSIS	62	0.60937	1.85012	0.001812	0.036503	0.031576
PID_IL8_CXCR1_PATHWAY	23	0.73136	1.81709	0.001949	0.036503	0.031576
WP_IL3_SIGNALING_PATHWAY	48	0.6214	1.80337	0.001859	0.036503	0.031576
KEGG_FC_EPSILON_RI_SIGNALING_PATHWAY	71	0.57231	1.78336	0.001795	0.036503	0.031576
REACTOME_INTERLEUKIN_4_AND_INTERLEUKIN_13_SIGNALING	97	0.54652	1.77469	0.001721	0.036503	0.031576
REACTOME_GLUCOSE_METABOLISM	81	0.55491	1.7502	0.001808	0.036503	0.031576
WP_IL18_SIGNALING_PATHWAY	234	0.45265	1.64082	0.001616	0.036503	0.031576

GSEA, Gene Set Enrichment Analysis.

### 3.6 PPI network

**Figure 6:**
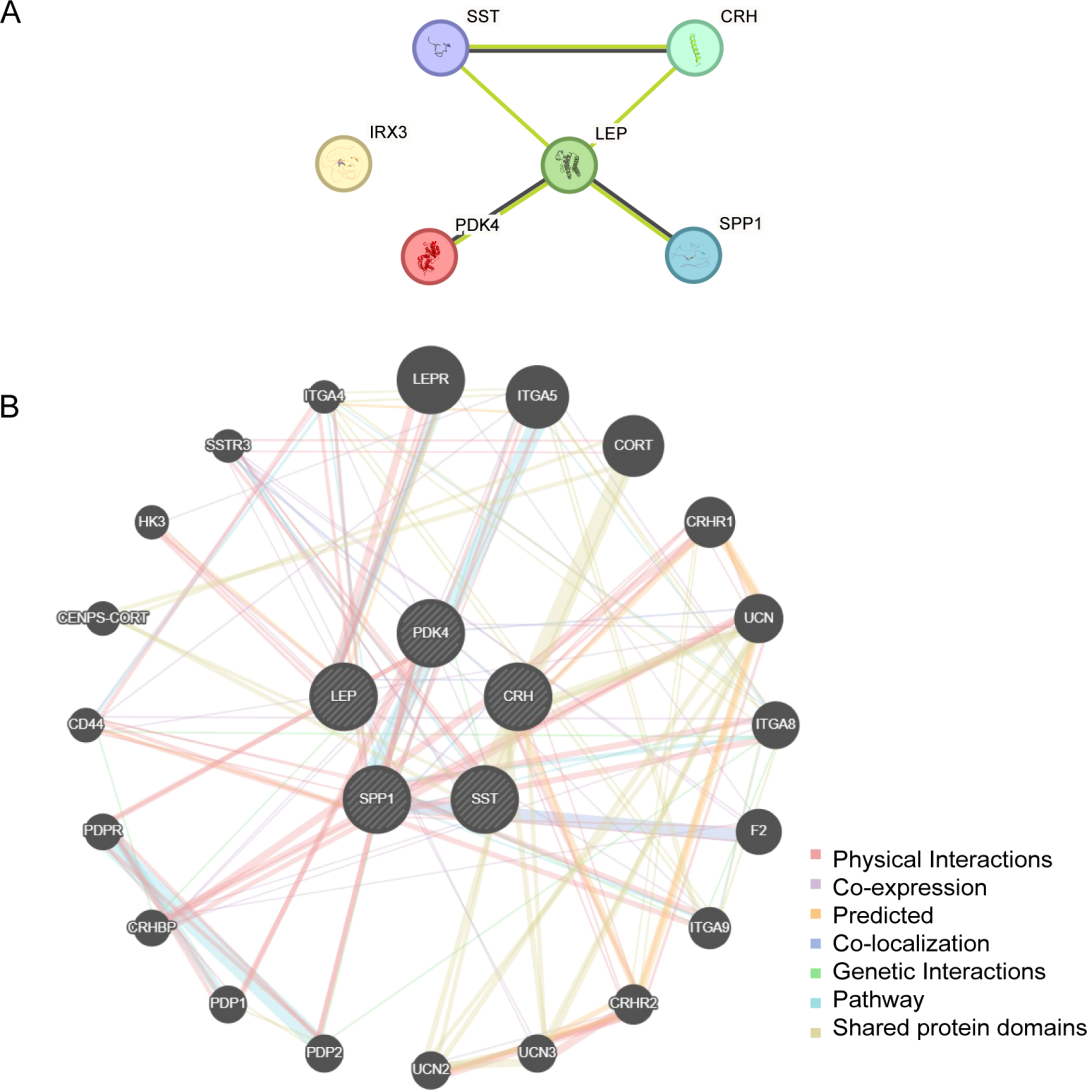
PPI network analysis. **(A)** PPI Network of EMRDEGs calculated from the STRING database. **(B)** The GeneMANIA website predicts the interaction network of functionally similar genes of EMRDEGs. The circles in the figure indicate the EMRDEGs and their functionally identical genes, and the corresponding colors of the lines represent the interconnected functions. PPI, Protein-protein Interaction; EMRDEGs, Energy Metabolism-Related Differentially Expressed Genes.

PPI interaction analysis was performed, and the PPI network of six EMRDEGs was constructed using the STRING database (**Figure 6A**). The PPI network findings indicated a connection among five EMRDEGs: *CRH*, *LEP*, *PDK4*, *SPP1*, and *SST*. Furthermore, the interaction network of five EMRDEGs and their functionally similar genes (**Figure 6B**) was predicted and constructed using the GeneMANIA website. The colored lines represent their co-expression and share protein domains and other information. Among them, there were 5 EMRDEGs and 20 functionally similar proteins.

### 3.7 Construction of regulatory network

**Figure 7:**
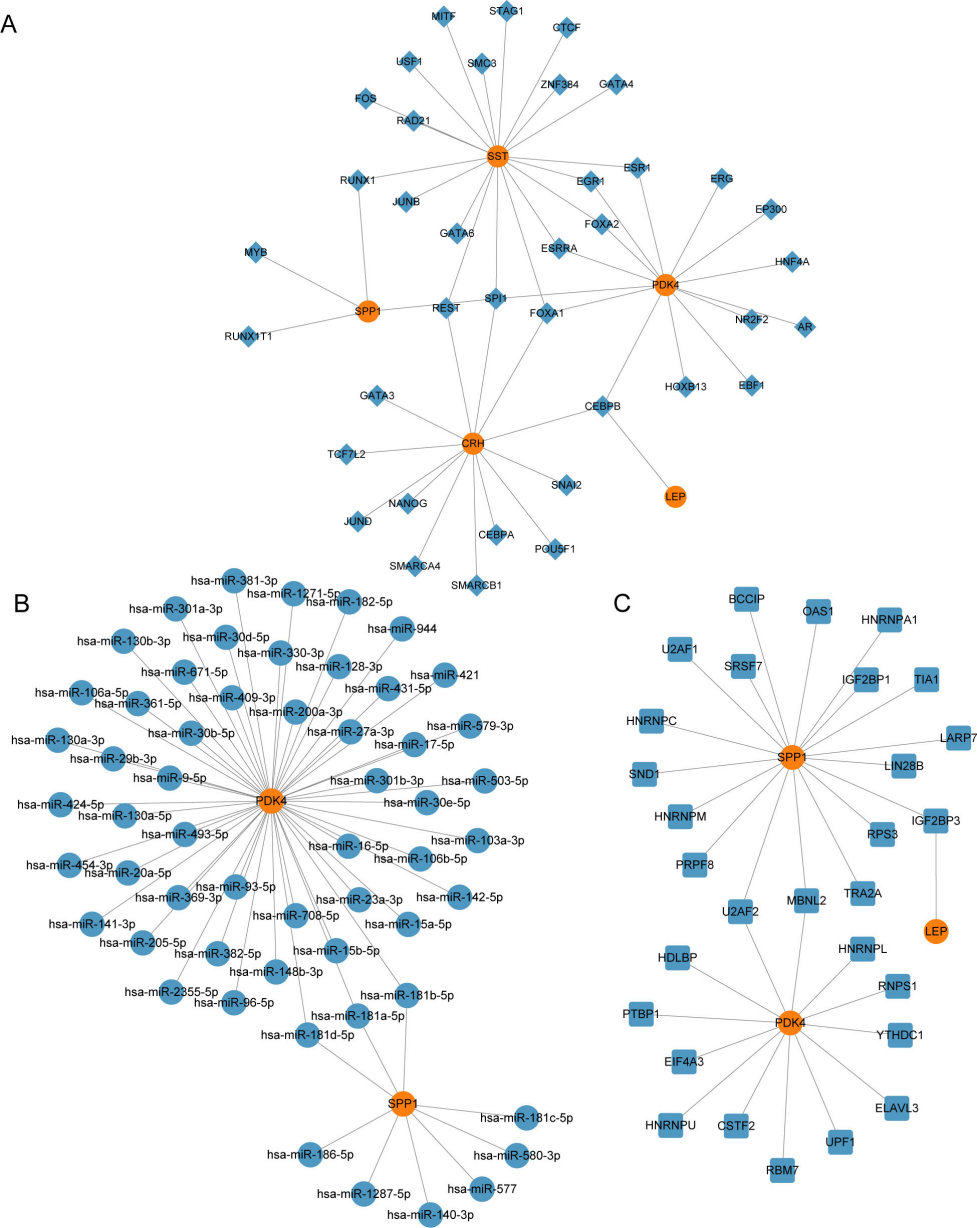
Regulatory network of EMRDEGs. **(A)** mRNA-TF Regulatory Network of Key Genes. **(B)** mRNA-miRNA Regulatory Network of Key Genes. **(C)** mRNA-RBP Regulatory Network of Key Genes. EMRDEGs, Energy Metabolism-Related Differentially Expressed Genes; TF, Transcription Factor; RBP, RNA-Binding Protein. Orange is mRNA, blue diamonds are TF, blue circles are miRNA, and blue squares are RBP.

We constructed the mRNA-TF regulatory network, which included five key genes (*CRH*, *LEP*, *PDK4*, *SPP1*, and *SST*) and 39 TFs, resulting in 51 mRNA-TF interactions (**Figure 7A**). Detailed information is provided in **Supplementary Table S2**. The mRNA-miRNA regulatory network consisted of two key genes (*PDK4* and *SPP1*) and 56 miRNAs, resulting in 59 mRNA-miRNA interactions (**Figure 7B**). Detailed data is presented in **Supplementary Table S3**. Our derived mRNA-RBP network included three key genes (*LEP*, *PDK4*, and *SPP1*) and 30 RBP molecules, resulting in 32 mRNA-RBP interactions (**Figure 7C**). Detailed data is provided be found in **Supplementary Table S4**.

### 3.8 Validation of differential gene expression and analysis of key genes using ROC curves

**Figure 8:**
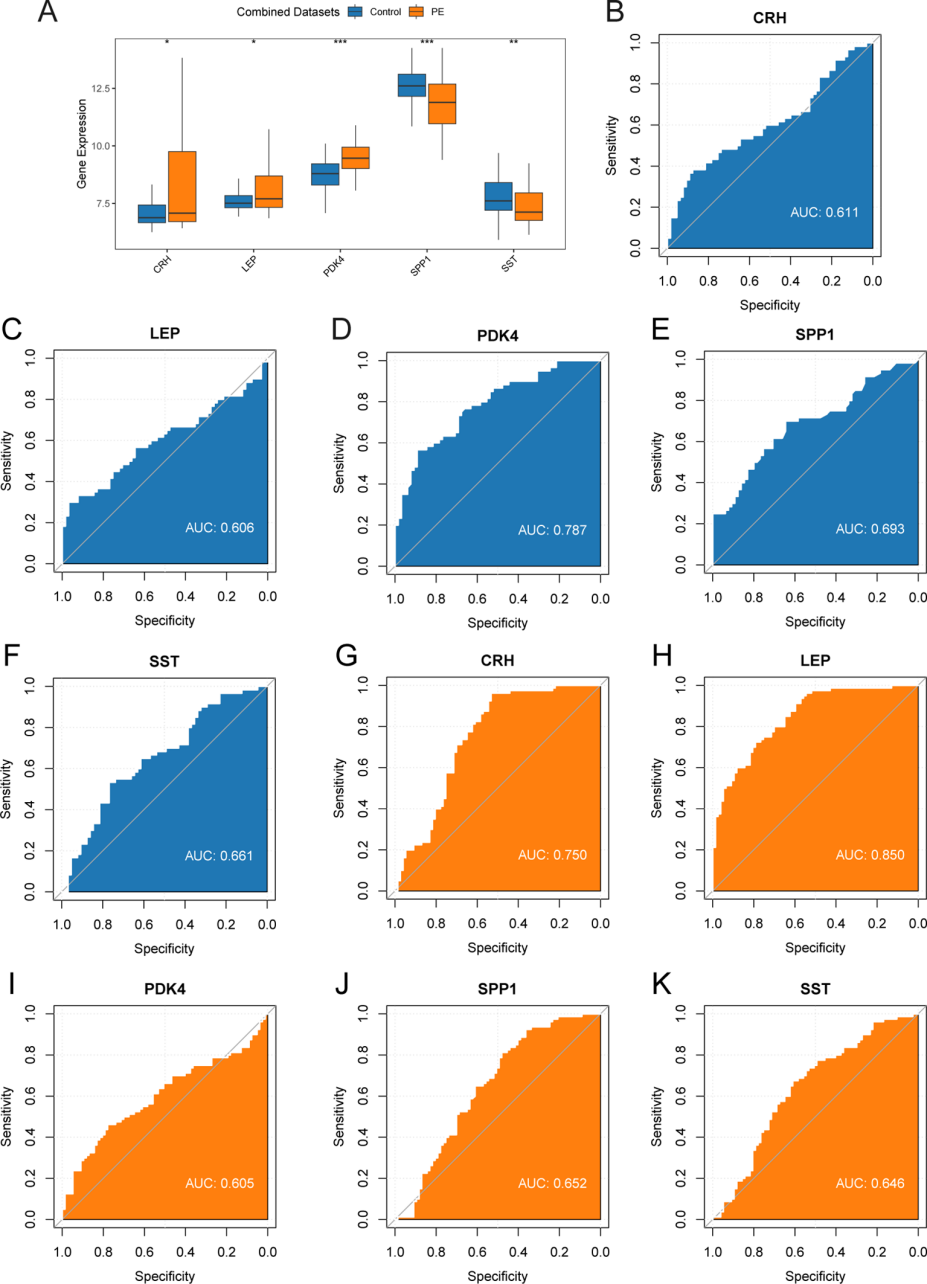
Differential expression validation and ROC curve analysis. **(A)** Group comparison plot of Key Genes in PE samples and Control samples of combined datasets. B-F. ROC curves of Key Genes *CRH* **(B)**, *LEP* **(C)**, *PDK4* **(D)**, *SPP1* **(E)**, and *SST* **(F)** in combined datasets. G-K. ROC curves of Key Genes *CRH* **(G)**, *LEP* **(H)**, *PDK4* **(I)**, *SPP1* **(J)**, and *SST* **(K)** in dataset GSE75010. ROC, Receiver Operating Characteristic; AUC, Area Under The Curve. ROC, Receiver Operating Characteristic Curve; TPR, True Positive Rate; FPR, False Positive Rate. * represents *p*-value < 0.05, statistically significant; ** represents *p*-value < 0.01, highly statistically significant; *** represents *p*-value < 0.001 and highly statistically significant. AUC between 0.5-0.7 had low accuracy, and AUC of 0.7-0.9 had moderate accuracy. In the group comparison figure, the PE group is orange, and the Control group is blue.

To investigate the key genes (*CRH*, *LEP*, *PDK4*, *SPP1*, and *SST*) across the combined datasets, a comparative analysis was performed through a group comparison (**Figure 8A**), indicating the outcomes of the differential expression analysis for these five key genes in PE samples compared with control samples from the combined datasets. The findings from the differential analysis (**Figure 8A**) indicated that two crucial genes (*PDK4* and *SPP1*) exhibited a significant statistical difference (*p* < 0.001) in both PE and control groups across the combined datasets. Furthermore, *SST* demonstrated a statistical significance (*p* < 0.01) across both types of samples. The other two crucial genes (*CRH* and *LEP*) exhibited significant expression in both PE and control groups, with a *p* < 0.05. Moreover, the expression levels of crucial genes in combined datasets were evaluated by creating ROC curves with the “pROC” package in R. The ROC curve (**Figures 8B–F**) revealed that the expression levels of key genes, including *PDK4* in PE samples, exhibited moderate to high accuracy across different groups (AUC: 0.7–0.9). The expression levels of crucial genes (*CRH*, *LEP*, *SPP1*, and *SST*) in PE samples demonstrated low precision across various groups (AUC: 0.5–0.7). The ROC curves for key genes in dataset GSE75010 (**Figures 8G–K**) revealed that the expression levels of *CRH* and *LEP* in PE samples demonstrated moderate to high precision among various groups (AUC between 0.7 and 0.9). Conversely, the expression levels of crucial genes (*PDK4*, *SPP1*, and *SST*) in PE samples demonstrated reduced precision across different groups (AUC: 0.5–0.7).

### 3.9 Immune infiltration analysis

**Figure 9:**
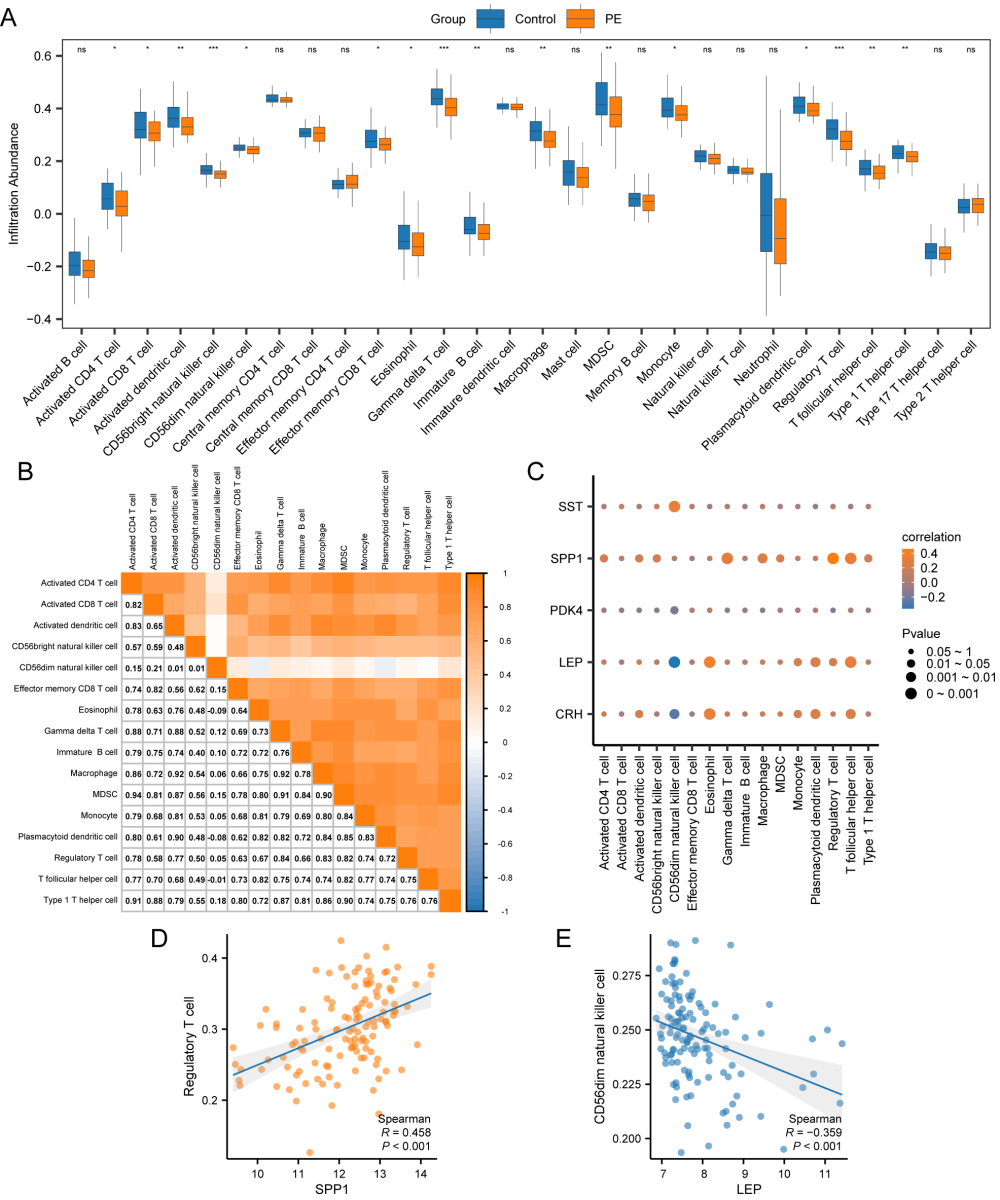
Immune infiltration analysis by ssGSEA algorithm. **(A)** Group comparison plot of immune cells in PE and Control samples from the combined datasets. **(B)** Correlation heatmap of immune cell infiltration abundance in the combined datasets. **(C)** Bubble correlation plot between Key Genes and immune cell infiltration abundance in the combined datasets. **(D)** Scatter plot of the correlation between Top1 positively correlated Key Genes and immune cells. **(E)** Scatter plot of correlation between TOP1-negatively correlated Key Genes and immune cells. ssGSEA, single-sample Gene-Set Enrichment Analysis; ns stands for *p*-value ≥ 0.05, not statistically significant; * represents *p*-value < 0.05, statistically significant; ** represents *p*-value < 0.01, highly statistically significant; *** represents *p*-value < 0.001 and highly statistically significant. The absolute value of the correlation coefficient (r-value) below 0.3 was weak or no correlation; between 0.3 and 0.5 was a weak correlation, between 0.5 and 0.8 was a moderate correlation, and above 0.8 was a strong correlation. In the group comparison plot, the PE samples are orange, and the Control samples are blue. In the heat map and correlation map, orange is a positive correlation, blue is a negative correlation, and the depth of color represents the strength of the correlation.

The ssGSEA algorithm evaluated the presence of 28 types of immune cells using expression data from combined datasets. Immune cells were selected with a *p* < 0.05 using a comparative group plot. The differences in immune cell infiltration levels across various groups were observed. The comparative chart (**Figure 9A**) indicateds that 16 immune cell types, including activated CD4+ T cells, activated CD8+ T cells, activated dendritic cells, CD56bright NK cells, CD56dim NK cells, effector memory CD8+ T cells, eosinophils, gamma-delta T cells, immature B cells, macrophages, myeloid-derived suppressor cells (MDSCs), monocytes, plasmacytoid dendritic cells, Tregs, T follicular helper cells, and type 1 T helper cells, exhibited significant differences between PE and control samples (*p* < 0.05).The correlation results from the combined datasets (**Figure 9B**) exhibited the abundance of 16 different immune cell infiltrations in the immune infiltration study. The results revealed a significant correlation among immune cells. The correlation between 5 key genes and 16 immune cells was examined and visualized using a correlation bubble diagram (**Figure 9C**). The results indicated a significant positive correlation between *SPP1* and Tregs, with an r-value = 0.458 and a *p* < 0.05. The key gene *LEP* exhibited a significantly negative correlation with CD56dim NK cells (r-value = –0.359, *p* < 0.05). Finally, a correlation scatter plot demonstrated the relationship between top1 positive and top1 negative key genes and immune cells (**Figures 9D, E**).

### 3.10 Validation of key genes in PE

**Figure 10:**
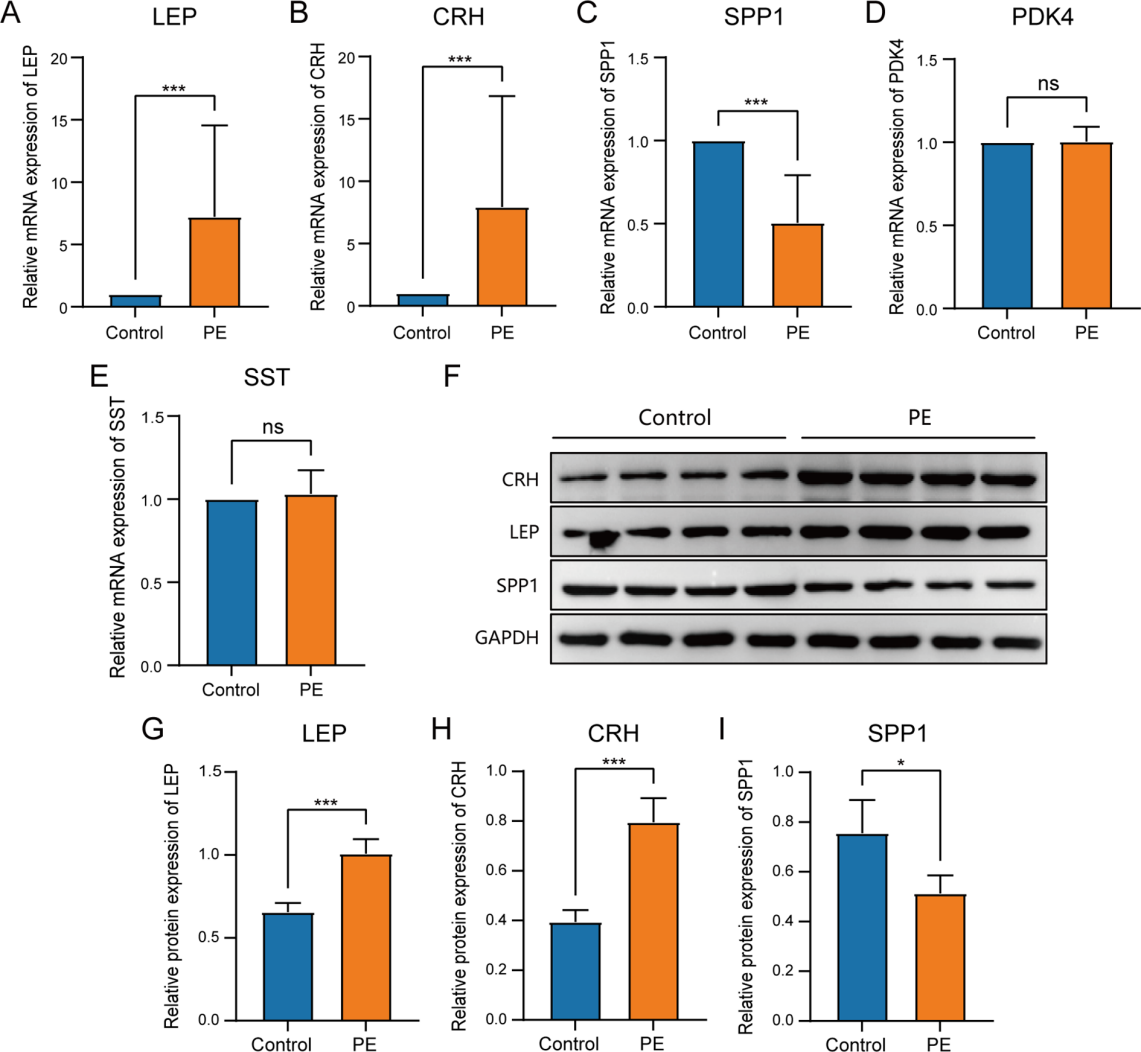
Comparison of key genes expression in placental samples of the control group and PE group. The expression bars of *LEP* **(A)**, *CRH* **(B)**, *SPP1* **(C)**, *PDK4* **(D)**, and *SST* **(E)** in the control group and PE group describe the mRNA expression levels of key genes. **(F)**Western blot analysis of *LEP*, *CRH*, *SPP1* protein expression levels in placental samples of preeclampsia group and control group. The expression bars of *LEP* **(G)**, *CRH* **(H)**, and *SPP1* **(I)** in the control group and PE group describe the protein expression levels of key genes.Blue bars representing the control group and orange representing the PE group. ns stands for *p*-value ≥ 0.05, not statistically significant; * represents *p*-value < 0.05, statistically significant; *** represents *p*-value < 0.001 and highly statistically significant.

To determine the mRNA expression levels of five crucial genes in PE, qRT-PCR analysis was performed on 26 patients with PE and 26 placental samples of comparable gestational age. **Table 5** presents the primer sequences. The clinical features of the patient are presented in **Table 6**. The two groups exhibited no significant variances in gestational age and birth weight. The PE group revealed higher systolic and diastolic blood pressure levels than the control group. The results of qRT-PCR indicated that, in contrast to the control group, the expressions of *LEP* and *CRH* in placental samples of PE patients were significantly elevated, while the expression level of *SPP1* was significantly reduced (**Figures 10A–E**). Through the Western blotting experiment, we further examined the protein expression levels of *LEP*, *CRH*, and *SPP1* (**Figures 10F**). The findings demonstrated that, compared with the control group, the expression of *LEP* and *CRH* proteins in placental samples of PE patients increased markedly, while the expression level of *SPP1* decreased conspicuously (**Figures 10G–I**).

**Table 5:** Primer sequences for qRT-PCR.

Gene	Primer sequences (5′-3′)
CRH	GGTCCCTACTCCTACTGCAAC (forward) CCAAGCATTCTCGATAGGCATTC (reverse)
LEP	TGCCTTCCAGAAACGTGATCC (forward) CTCTGTGGAGTAGCCTGAAGC (reverse)
PDK4	GACCCAGTCACCAATCAAAATCT (forward) GGTTCATCAGCATCCGAGTAGA (reverse)
SPP1	CTCCATTGACTCGAACGACTC (forward) CAGGTCTGCGAAACTTCTTAGAT (reverse)
SST	ACCCAACCAGACGGAGAATGA (forward) ACCCAACCAGACGGAGAATGA (reverse)

qRT−PCR, quantitative real−time PCR.

**Table 6:** Clinical information of the patients.

Category	PE (n = 26)	Control (n = 26)	*p*-value
Age (years)	31.385 ± 5.93	29.846 ± 4.62	0.302
Gestational age at delivery (weeks)	36.423 ± 2.53	36.423 ± 2.53	1
Systolic blood pressure(mmHg)	155.73 ± 20.36	121.38 ± 6.84	< 0.001
Diastolic blood pressure (mmHg)	93.654 ± 10.88	74.346 ± 4.09	< 0.001
Neonatal birth weight (g)	2713.8 ± 618.71	2934.6 ± 576.38	0.323
1 min Apgar (score)			0.204
10	23 (88.5%)	26 (100%)	
9	2 (7.7%)	0 (0%)	
7	1 (3.8%)	0 (0%)	
5 min Apgar (score)			0.471
10	24 (92.3%)	26 (100%)	
9	2 (7.7%)	0 (0%)	

## 4 Discussion

PE is a complicated disorder that impacts around 2%–8% of pregnancies globally and continues to be a major contributor to maternal and perinatal illness and death (36). PE occurs after the 20^th^ week of pregnancy and is characterized by the sudden onset of high blood pressure and protein in the urine. PE can result in serious complications, including eclampsia, HELLP syndrome, and long-term cardiovascular risks for both the mother and child (37). The exact mechanisms underlying PE remain unclear; however, it is hypothesized that irregular placental growth and operation cause widespread inflammation and impairment of endothelial function (38). Current diagnostic approaches for PE primarily depend on blood pressure monitoring and urinalysis for proteinuria. Nevertheless, these methodologies are often nonspecific and can only detect the disease in its advanced stages, which leads to delayed intervention (39). Research into the underlying mechanisms of PE is imperative for the development of predictive biomarkers and effective therapeutic strategies, as it has a substantial health impact on pregnant women and their offspring.

Currently, the diagnosis of PE often relies on multiple biomarkers, with the most common being *soluble vascular endothelial growth factor receptor-1* (*sFlt-1*) and *placental growth factor* (*PlGF*). *sFlt-1* is an anti-angiogenic factor secreted by the placenta, with significantly elevated levels in PE patients, while *PlGF* is a factor that promotes blood vessel formation and generally exhibits decreased levels during PE. Research has demonstrated that the *sFlt-1*/*PlGF* ratio can serve as an effective indicator for assessing both the occurrence and severity of PE (40). By detecting this ratio, clinicians can identify PE at an early stage, thereby providing a foundation for timely intervention. However, despite their potential application in diagnosing PE, *sFlt-1* and *PlGF* possess limited accuracy and specificity. Firstly, these markers’ levels are not only influenced by PE but may also be affected by other pregnancy-related factors such as gestational diabetes or placental abruption, leading to false positive or negative results. Furthermore, fluctuations in *sFlt-1* and *PlGF* levels throughout pregnancy may impact measurements at a single time point. Therefore, relying solely on these two biomarkers is insufficient for definitively diagnosing PE; integrating additional biomarkers may enhance diagnostic precision.

New developments in genomics and bioinformatics have created opportunities to understand the molecular foundations of diseases, including PE. By integrating phenotypic data with high-throughput molecular analyses, researchers can reveal novel biomarkers and therapeutic targets that can revolutionize PE management (41). Recent studies have suggested that alterations in EMRGs can contribute to PE pathogenesis by affecting placental function and maternal systemic response (42). This research direction holds the potential to improve our understanding of PE as well as identify novel diagnostic markers that can result in earlier detection and intervention, thereby improving outcomes for mothers and their offspring (43). Significant progress has been achieved; however, there is a crucial lack in our comprehension of the complex molecular mechanisms contributing to PE development. These gaps underscore the necessity for further investigation.

In this study, we have identified six EMRDEGs and observed their differential expression in patients with PE. These genes may reflect the metabolic and immune changes occurring in PE, offering novel diagnostic insights. Our findings underscore the significance of metabolic alterations in the pathogenesis of PE, a facet that has been underappreciated among existing biomarkers such as *sFlt-1* and *PlGF*. Furthermore, variations in EMRDEGs may offer new perspectives into the pathophysiological mechanisms underlying PE and complement ongoing efforts to identify early diagnostic markers.

Our findings indicated a significant upregulation of *LEP* in placental samples from patients with PE, suggesting that dysregulation of the *LEP* gene could contribute to the metabolic disturbances observed in PE. *LEP* emerged as a critical player among the DEGs. *LEP*, an adipose tissue-derived hormone, controls energy equilibrium and metabolic processes (44). Increased *LEP* levels have been associated with insulin resistance and inflammation (45), both of which are pertinent in the context of PE. Additionally, prior research has indicated the effect of *LEP* on regulating vascular function and its potential role in the onset of hypertension, a characteristic feature of PE (46). Consequently, the upregulation of *LEP* in PE can reflect an adaptive response to altered energy metabolism, consistent with our findings.

*SPP1* is another key gene that exhibited significant downregulation in our analysis. *SPP1*, an osteopontin, plays a role in multiple biological activities, including cell adhesion, movement, and immune response regulation (47). Its expression is crucial for placental development and function. Its reduced expression in PE could impair trophoblast invasion and placental development, resulting in inadequate remodeling of maternal spiral arteries (48), which was validated by our study.

Furthermore, *CRH* was identified as a DEG in our study. *CRH* is an important neuropeptide whose secretion is regulated by a variety of factors, including physiological and environmental stress. Studies have shown that during pregnancy, the synthesis and secretion of *CRH* increases significantly, which is closely related to pregnancy-related physiological changes. In particular, during the second and third trimesters of pregnancy, the level of *CRH* in the maternal blood increases significantly, which may be due to the synthetic effects of the placenta. The placenta regulates the maternal immune response and endocrine system by producing *CRH*, and affects the blood flow and nutrient supply of the placenta, which may lead to placental dysfunction and hypertension [16]. Collectively, these findings improve our understanding of the molecular foundations of PE and open new avenues for targeted interventions to restore normal energy metabolism and placental function.

Our study identified several EMRGs that exhibited differential expression in PE, focusing on their involvement in glycolysis and gluconeogenesis pathways. Glycolysis and gluconeogenesis are critical metabolic pathways that regulate glucose homeostasis, providing energy and metabolic intermediates for various cellular processes. The dysregulation of these pathways can result in altered energy metabolism, a hallmark of PE.

Recent studies have suggested the importance of metabolic alterations in PE pathogenesis, demonstrating a shift towards glycolytic metabolism as a potential disease hallmark. Ackerman et al. observed a glycolytic change of placental tissues from cases of early-onset PE with or without fetal growth restriction, pointing to altered tissue bioenergetics (49). Hu et al. further reinforced the idea by examining exosomal mRNA and lncRNA profiles in cord blood and identifying the involvement of glycolysis and gluconeogenesis in developing PE (50). In line with our GSEA, which identified significant enrichment of the glycolysis and gluconeogenesis pathways in PE, this underscores the critical role of metabolic changes in the pathophysiology of diseases.

Moreover, the differential expression of EMRGs in PE suggested a broader impact on cellular energy metabolism and oxidative stress. Li et al. identified key proteins associated with glycolysis/gluconeogenesis and oxidative phosphorylation in syncytiotrophoblast extracellular vesicles from patients with early-onset severe PE, further supporting the role of disrupted energy metabolism in the condition (51). Furthermore,Tong et al. found that genes involved in glycolysis/gluconeogenesis were significantly inhibited in the decidua of severe PE, indicating impaired energy metabolism at the maternal-fetal interface (52).

Meanwhile, GO and KEGG analyses in the study highlighted crucial roles in lipid metabolism, hormone function, and bone remodeling. The varying expression levels of genes, including *CRH*, *LEP*, *PDK4*, *SPP1*, and *SST*, provided evidence for possible biomarkers and treatment targets. The alteration in *LEP* levels, known for regulating energy homeostasis, underscored the complex interplay of metabolic disruptions in PE. Furthermore, creating networks for protein interactions and regulatory frameworks involving TFs, miRNAs, and RBPs provided a deeper insight into the molecular interactions and regulatory processes in PE. The findings highlighted the complexity of gene regulation and the potential for targeted therapeutic interventions.

The immune landscape in PE is characterized by a complex interplay of altered innate and adaptive immune responses, which is crucial for understanding disease pathogenesis. Zhou et al. identified shifts in NK cell gene expression and an increase in Tregs in PE via single-cell RNA sequencing, suggesting a response to counteract the inflammatory state of PE (53). Han et al.’s employed mass cytometry to analyze maternal blood and depicted immune feature shifts that predicted PE, with early pregnancy marked by a proinflammatory response and diminished Treg signaling, highlighting the role of early immune dysregulation in PE (54). Furthermore, Luo et al.focused on the dysfunction of NK cells and macrophages, demonstrating that aberrant human leukocyte antigen (HLA) molecule expression by extravillous trophoblasts could enhance NK cell cytotoxicity, exacerbating placental dysfunction (55). Our research confirmed the preceding findings, revealing significant alterations in different immune cells, including Tregs and CD56dim NK cells, in samples from patients with PE compared to the control group. These findings underscored the importance of immune cells in preserving immune tolerance at the maternal-fetal interface, highlighting the necessity for additional research on how these immune alterations impacted the advancement and detection of the disease.

An imbalance in energy metabolism significantly influences the immune system through various mechanisms, aligning with our findings on the interactions between specific genes and immune cell populations in PE. Firstly, insulin resistance and abnormal lipid metabolism can trigger systemic inflammation, leading to elevated levels of pro-inflammatory cytokines such as TNF-α and IL-6 (56). These cytokines activate immune cells and alter their function and distribution, contributing to the immune dysregulation observed in PE. Secondly, energy metabolism imbalances can directly affect the metabolic pathways of immune cells; for example, changes in glucose metabolism can impact the activity and function of T cells and macrophages (57). Furthermore, metabolic dysregulation can impact the functionality of immune cells such as NK cells and Tregs, resulting in the breakdown of immune tolerance and exacerbation of inflammatory response, thereby further worsening the immune dysfunction observed in PE (58). These mechanisms underscore the intricate relationship between energy metabolism and immune regulation, highlighting the potential of targeting metabolic pathways to modulate immune responses in PE.

Our findings underscored significant interactions between certain genes and specific immune cell populations, offering insights into the intricate immune modulation in PE. Furthermore, we observed a significant positive correlation between *SPP1* and Tregs. The gene *SPP1* is recognized for its involvement in regulating the immune system, promoting blood vessel formation, and modifying tissue structure (59). However, no direct studies have been published on the association between *SPP1* and Tregs in PE, *SPP1* is among the most upregulated genes during T-cell activation, and it has diverse roles in immune response regulation (60). This relationship suggests that *SPP1* promotes an immunosuppressive environment conducive to fetal tolerance. Additionally, Tregs are known for maintaining immune homeostasis and preventing autoimmunity by suppressing abnormal immune responses. Based on the established mechanisms, we hypothesized that reduced expression of *SPP1* in PE could negatively impact the immunosuppressive environment and the expansion and function of Tregs, ultimately affecting overall immune homeostasis and potentially contributing to the pathophysiological processes of PE.

Furthermore, our research exhibited a negative correlation between *LEP* and CD56dim NK cells, highlighting the intricate interplay between the *LEP* gene and specific NK cell subsets. This subset, well-known for its crucial role in regulating maternal-fetal immunity, is influenced by the signaling pathways of *LEP* (61). NK cells are critical in establishing appropriate maternal-fetal immune interactions in early pregnancy, indicating the complex role of *LEP* in regulating adverse immune responses in PE. Increased *LEP* levels, possibly reflective of the inflammatory state and placental insufficiency in PE, may impair the cytolytic function of CD56dim NK cells, hampering their role in placental and fetal development. Moreover, *LEP* emerges as a potentially influential factor in shaping the immune environment in PE, potentially impacting the balance between tolerance and immune activation necessary for a successful pregnancy outcome. In conclusion, the immune system dysfunction observed in PE, which is marked by alterations in the infiltration and activity of immune cells, highlights the significance of immune processes in disease pathophysiology. These findings provided valuable insights into potential therapeutic targets and highlighted the need for further research to develop immune-based interventions for PE.

The potential of biomarkers such as *LEP*, *SPP1*, and *CRH* in predicting the severity or complications of PE is noteworthy. Differential expression analysis from the combined datasets revealed significant differences in the expression of these biomarkers between PE and control groups. This was confirmed by qRT-PCR and Western blot of clinical samples. Additionally, the correlation between key genes and specific immune cells indicates that these biomarkers may modulate immune responses in PE. Given their significant differential expression and association with energy metabolism and immune regulation, these key genes hold promise as biomarkers for predicting PE severity or complications.

The identified biomarkers were compared with established diagnostic markers for PE, such as *sFlt-1* and *PlGF*. The mechanism of *sFlt-1* involves inhibiting normal angiogenesis, leading to placental dysfunction and symptoms like hypertension, while *PlGF* levels typically decrease in PE patients (41). The *sFlt-1*/*PlGF* ratio is widely used for early PE diagnosis due to its high specificity and sensitivity. In contrast, *LEP* is involved in energy balance and metabolic processes, with its upregulation in PE placental samples potentially linked to insulin resistance and inflammation. The increase of CRH level in PE patients may be related to the disturbance of placental energy metabolism, and further aggravate the condition by affecting placental oxidative stress and inflammatory response. *SPP1* downregulation may affect trophoblast invasion and placental development. While *sFlt-1* and *PlGF* are well-established in clinical practice for early identification of high-risk pregnancies, *LEP*, *SPP1*, and *CRH* hold potential as new diagnostic and therapeutic targets. Further research on these new biomarkers could enhance our understanding of PE’s underlying mechanisms and improve diagnostic and treatment strategies.

Known treatments for PE, such as antihypertensive drugs, early low-dose aspirin, and calcium supplements, may influence the expression of the identified genes. For instance, antihypertensive drugs have been shown to affect *LEP* expression by improving blood flow and reducing blood pressure (62). Early low-dose aspirin may regulate *LEP* levels by inhibiting platelet activation and associated inflammatory responses, thus improving energy metabolism and balance (63). These therapeutic interventions highlight the potential for targeted treatments to modulate gene expression and improve outcomes for patients with PE.

In light of our findings, exploring therapeutic approaches targeting the identified genes could offer new avenues for PE treatment. For instance, *LEP* ‘s therapeutic potential could be harnessed through anti-inflammatory drugs. Given the relationship between *LEP* and inflammatory states, certain anti-inflammatory medications, such as non-steroidal anti-inflammatory drugs (NSAIDs), might mitigate the inflammatory response in PE. By reducing *LEP* levels, these drugs could potentially improve maternal metabolic status and vascular function. Additionally, *SPP1*’s role in placental development and function suggests that therapies promoting *SPP1* expression could be beneficial. Furthermore, *SPP1*’s involvement in immune regulation indicates its potential as an immunomodulatory agent in PE treatment, promoting fetal tolerance and improving pregnancy outcomes.

Translating these findings into clinical practice presents several challenges. One major challenge is the standardization of biomarker assays. To effectively utilize new biomarkers such as *LEP*, *SPP1*, and *CRH* in clinical settings, standardized detection protocols must be established, including standardized detection methods and quality control measures. Additionally, clinical validation and external validation are crucial. Although our study has identified potential biomarkers, their clinical efficacy needs to be confirmed through large-scale clinical validation. This includes addressing challenges related to the scale and representativeness of the study population and the complexity of clinical scenarios.

Despite the comprehensive bioinformatics approach employed, this study has certain limitations. First, the quality and source of the data used can significantly impact the results, as variations in sample collection, processing, and storage conditions across different studies can introduce inconsistencies. Second, integrating multiple datasets from different sources could introduce batch effects despite efforts to correct these effects using computational methods. Such batch effects could still influence the results and interpretations. Third, the choice of analysis tools and algorithms can affect the outcomes, as different tools and algorithms may yield varying results, introducing bias. Fourth, functional annotation relies on existing databases, which may not be comprehensive or up-to-date, limiting the accuracy and completeness of the functional insights derived. Fifth, the study lacked extensive clinical validation, which was crucial for translating the findings into clinical practice, and the results need to be validated in larger, independent cohorts and through functional experiments to establish their clinical relevance. Additionally, the sample size was relatively small, with only 12 placental samples collected for qRT-PCR validation, which could limit the generalizability of the results. Lastly, the study was not conducted in cells or animals and was not robust enough to be validated in wet laboratory experiments, limiting the ability to confirm the biological significance of the identified genes and pathways.

## 5 Conclusion

In conclusion, the study effectively detected EMRDEGs in PE by integrating and analyzing various datasets. The functional enrichment studies indicated important BPs and pathways associated with EMRDEGs, offering an understanding of the fundamental mechanisms of PE. Identifying interactions between proteins and regulatory networks, including mRNA-TF, mRNA-miRNA, and mRNA-RBP, highlighted significant genes and their possible regulatory mechanisms. Immune infiltration analysis suggested that specific immune cell types were differentially abundant between PE and control groups and might correlate with key genes. The potential for diagnosis using these important genes was revealed through ROC curves, and their expression was confirmed using qRT-PCR. The results provided a comprehensive insight into the molecular foundation of PE and indicated possible biomarkers and targets for treatment. Future studies should focus on larger sample sizes, wet lab validations, and extensive clinical trials to further substantiate the findings and facilitate their translation into clinical applications.

## Data Availability

The original contributions presented in the study are included in the article/**Supplementary Material**. Further inquiries can be directed to the corresponding author/s.

## Supplementary material

Supplementary Table 1 EMRGs.

Supplementary Table 2 mRNA-TF.

Supplementary Table 3 mRNA-miRNA.

Supplementary Table 4 mRNA-RPB.
